# 头孢他啶阿维巴坦治疗血液病患者粒细胞缺乏期感染的疗效分析

**DOI:** 10.3760/cma.j.issn.0253-2727.2022.12.011

**Published:** 2022-12

**Authors:** 欣 孔, 剑 张, 梦云 郦, 爱宁 孙, 悦 韩, 晓文 唐, 惠英 仇, 德沛 吴

**Affiliations:** 苏州大学附属第一医院血液科，国家血液系统疾病临床医学研究中心，江苏省血液研究所，卫生部血栓与止血重点实验室，苏州 215006 National Clinical Research Center for Hematologic Diseases, Jiangsu Institute of Hematology, Department of Hematology, The First Affiliated Hospital of Soochow University, Suzhou 215006, China

碳青霉烯耐药革兰阴性菌（CRO）感染是血液病患者的重要威胁。CRO感染的治疗选择非常有限，目前临床已用方案包括多黏菌素E、多黏菌素B、黏菌素、头孢他啶阿维巴坦（CAZ-AVI）、替加环素、大剂量碳青霉烯类及磷霉素等。《中国成人医院获得性肺炎与呼吸机相关性肺炎诊疗指南（2018年版）》中推荐CAZ-AVI用于治疗碳青霉烯耐药肠杆菌（CRE）感染[1]。本研究中，我们回顾性分析了CAZ-AVI治疗粒细胞缺乏（粒缺）期感染疗效及安全性，现报道如下。

## 病例与方法

1. 临床资料：本研究为回顾性研究，以苏州大学附属第一医院自2019年9月至2022年8月血液病合并粒缺期感染且应用CAZ-AVI治疗的187例患者为研究对象。感染诊断依据卫生部颁布的《医院感染诊断标准（试行）》[2]，粒缺定义为外周血中性粒细胞绝对计数（ANC）<0.5×10^9^/L或预计48 h后ANC<0.5×10^9^/L；严重粒缺指ANC<0.1×10^9^/L[3]。

2. 病原菌分离、鉴定：187例患者在应用CAZ-AVI前均进行外周血需氧菌和厌氧菌培养、导管血需氧菌培养、咽拭子/痰培养、肛拭子/肛周分泌物培养、皮肤/会阴/软组织感染分泌物培养等，收集分离菌株鉴定信息及耐药率数据（同一患者14 d内只纳入第1次阳性结果），根据美国临床实验室标准化委员会（CLSI M100）2019年标准对主要病原菌进行药敏分析与判读，使用vitek 2 Compact 60进行配套的细菌鉴定及药敏试验。根据分离病原菌株的鉴定结果将患者分为：CRO组（分离出CRO菌株）、非CRO组（分离出非CRO菌株）、经验治疗组（未分离出病原菌），同时分离出CRO及非CRO病原菌者归入CRO组，碳青霉烯类天然耐药菌归入CRO组。CAZ-AVI药敏试验采用药敏纸片扩散法，药敏结果判读抑菌圈≥21 mm为敏感。

3. CAZ-AVI给药方案：CAZ-AVI 2.5 g，每8 h 1次，静脉滴注；体温正常5～7 d后予抗菌药物降阶梯治疗或停药。86例患者单用CAZ-AVI抗感染；101例患者为联合用药，联用药物包括替加环素、多黏菌素B、多黏菌素E、氨曲南、阿米卡星、美罗培南、头孢哌酮舒巴坦。

4. 统计学处理：采用SPSS 22.0统计软件进行数据分析。连续变量不符合正态分布，以“中位数（范围）”进行描述，采用Kruskal-Wallis *H*检验进行组间比较；计数资料采用“例数（百分比）”表示，采用*χ*^2^检验或校正*χ*^2^检验进行组间比较，*P*<0.05为差异有统计学意义。

## 结果

一、临床与病原学特征

187例血液病患者中位年龄42（14～74）岁，男女比为2.12∶1。其中，急性白血病145例，骨髓增生异常综合征12例，恶性淋巴瘤18例，重型再生障碍性贫血6例，多发性骨髓瘤3例，慢性白血病3例。158例为严重粒缺（84.0％）。全部187例患者均置入中心静脉导管。

122例血液病患者分离出致病菌138株，包括肺炎克雷伯杆菌44株、铜绿假单胞菌19株、大肠埃希菌18株、嗜麦芽窄食单胞菌18株、阴沟肠杆菌11株、新洋葱伯克霍尔德菌7株、其他21株。经药敏试验鉴定出CRO 80株［46株（33.3％）为CRE］、非CRO 58株，检出主要病原菌分布及构成比见表1，部分患者出现同一致病菌多部位检出，原因考虑血流感染播散至多脏器以及局部感染入血造成血流感染可能等。1例同时检出碳青霉烯类耐药肺炎克雷伯杆菌和碳青霉烯类耐药大肠埃希菌，7例CRO合并非CRO感染，分在CRO组；5例同时感染两种非CRO，1例同时感染4种非CRO，分在非CRO组。另65例患者未分离出致病菌，分在经验治疗组，其中6例有明显皮肤、肛周、会阴感染灶，29例影像学检查提示肺部感染，2例颅内感染，1例阑尾炎行手术治疗确诊，27例感染灶不明确。CRO组、非CRO组、经验治疗组患者的临床特征比较见表2，经验治疗组中位年龄明显低于CRO组与非CRO组，余临床特征三组差异均无统计学意义。

**表1:** 122例血液病患者中检出主要病原菌分布及构成比（株数）

病原菌种类	标本来源			检出数	CRO	CRE
	血培养	咽拭子/痰培养	肛拭子/肛周分泌物			
肺炎克雷伯杆菌	24	9	11	44	33	33
铜绿假单胞菌	12	6	2	19	12	0
大肠埃希菌	12	4	3	18	6	6
嗜麦芽窄食单胞菌	11	8	0	18	18	0
阴沟肠杆菌	2	8	2	11	4	4
新洋葱伯克霍尔德菌	2	5	1	7	0	0
皮氏不动杆菌	0	5	0	5	3	0
鲍曼不动杆菌	1	3	1	4	1	0
产气肠杆菌	2	0	0	2	2	2
产酸克雷伯杆菌	2	1	0	2	1	1
污染伯克霍尔德菌	0	1	0	1	0	0
其他^a^	3	2	0	7	0	0

合计	71	52	20	138	80	46

注 CRO：碳青霉烯耐药革兰阴性菌；CRE：碳青霉烯耐药肠杆菌；^a^其他：包括摩根菌、琼氏不动杆菌、海藻希瓦菌、黏金黄杆菌、丙二酸盐克洛诺杆菌、脑膜浓毒性伊丽莎白金菌、奇异变形杆菌

**表2:** 碳青霉烯耐药革兰阴性菌（CRO）、非CRO与经验治疗组粒细胞缺乏伴感染血液病患者临床特征比较

临床特征	CRO组（79例）	非CRO组（43例）	经验治疗组（65例）	统计量	*P*值
中位年龄［岁，*M*（范围）］	45（17~71）	46（17~68）	38（14~74）	9.00	0.01
男性［例（％）］	56（70.9）	25（58.1）	46（70.8）	2.45	0.29
疾病类型				5.29	0.87
急性白血病［例（％）］	62（78.5）	32（77.4）	51（78.5）		
骨髓增生异常综合征［例（％）］	6（7.6）	3（7.0）	3（4.6）		
非霍奇金淋巴瘤［例（％）］	6（7.6）	6（14.0）	6（9.2）		
重型再生障碍性贫血［例（％）］	3（3.8）	0	3（4.6）		
多发性骨髓瘤［例（％）］	1（1.3）	1（2.3）	1（1.5）		
慢性白血病［例（％）］	1（1.3）	1（2.3）	1（1.5）		
严重粒细胞缺乏［例（％）］	71（89.9）	36（83.7）	51（78.5）	3.61	0.17

二、药敏分析

排除感染两种及以上致病菌患者，对其主要革兰阴性菌进行常见抗菌药物敏感耐药性分析，结果见表3。肺炎克雷伯杆菌碳青霉烯类抗菌药物耐药率达74.4％，CAZ-AVI耐药率为27.8％。铜绿假单胞菌碳青霉烯类抗菌药物耐药率为56.3％，CAZ-AVI均敏感，大肠埃希菌CAZ-AVI耐药率为66.7％，高于碳青霉烯类耐药率（50.0％），部分为产金属酶菌株。共29例患者进行CAZ-AVI药敏试验，结果提示22例敏感（75.9％），7例原发耐药，耐药率为24.1％。5例碳青霉烯类耐药大肠埃希菌患者中2例进行CAZ-AVI药敏试验，均提示原发耐药。因条件限制本中心未做细菌产酶鉴定。

**表3:** 主要革兰阴性菌对常见抗菌药物耐药率（％）

抗菌药物	肺炎克雷伯菌（*n*＝39）	阴沟肠杆菌（*n*＝9）	铜绿假单胞菌（*n*＝16）	大肠埃希菌（*n*＝10）	鲍曼不动杆菌（*n*＝5）	嗜麦芽窄食单胞菌（*n*＝11）	洋葱伯克霍尔德菌复合群（*n*＝6）
头孢曲松	100.0	55.6	–	100.0	80.0	–	–
头孢他啶	100.0	55.6	6.3	80.0	100.0	36.4	0.0
头孢吡肟	86.8	44.4	6.3	90.0	100.0	–	–
头孢哌酮舒巴坦	84.2	44.4	12.5	90.0	0.0	36.4	50.0
哌拉西林他唑巴坦	89.5	55.6	31.3	80.0	80.0	–	–
头孢他啶阿维巴坦	27.8	–	0.0	66.7	–	–	–
亚胺培南	74.4	33.3	81.3	50.0	80.0	–	–
美罗培南	79.5	33.3	56.3	50.0	80.0	–	0.0
阿米卡星	30.8	0.0	6.3	10.0	20.0	–	–
左氧氟沙星	92.3	55.6	18.8	90.0	20.0	27.3	0.0
复方磺胺甲恶唑	71.8	55.6	–	100.0	80.0	27.3	16.7

注 –：天然耐药未做药敏试验

三、疗效及不良反应

5例检出碳青霉烯类耐药大肠埃希菌患者中3例死亡，均在未取得菌株鉴定结果前死亡；1例经验性联用氨曲南，后药敏鉴定提示氨曲南亦耐药。

CRO组79例，其中CRE定植筛查阳性者12例，随着粒缺时间延长均转为CRE感染，在出现感染症状后调整为CAZ-AVI抗感染；非CRO组43例，其中替加环素治疗无效26例，碳青霉烯类治疗无效14例，头孢菌素治疗无效3例。经验治疗组65例，替加环素治疗无效51例，碳青霉烯类治疗无效14例。CRO组、非CRO组、经验治疗组存在危及生命的血液动力学不稳定病例数分别为23例（29.1％）、11例（25.6％）、18例（27.7％）。

187例患者应用CAZ-AVI治疗后，临床有效133例，无效54例，总体治疗有效率为71.1％，28 d全因累积死亡率为19.8％（37/187）。133例有效患者体温恢复正常中位时间为1.5（0.3～11）d。CRO组、非CRO组、经验治疗组患者治疗有效率分别为65.8％（52/79）、74.4％（32/43）、75.4％（49/65）（*χ*^2^＝1.88，*P*＝0.39），28 d全因累积死亡率分别为20.3％（16/79）、20.9％（9/43）、18.5％（12/65）（*χ*^2^＝0.12，*P*＝0.94）。三组间有效患者体温恢复正常的中位时间分别为1.5（0.3～11）、1.0（0.3～3.9）、1.6（0.3～6.5）d（*H*＝3.65，*P*＝0.16）。CRO组与非CRO组7 d微生物学清除率分别为57.0％（45/79）对69.8％（30/43），差异无统计学意义（*χ*^2^＝1.928，*P*＝0.165）。

CAZ-AVI单药应用86例，有效66例，治疗有效率76.7％，19例用药时已存在血流动力学不稳定。联合其他抗菌药物应用101例，有效67例，治疗有效率为66.3％，33例用药时已存在血流动力学不稳定。联用药物方案中，与替加环素联用29例，治疗有效率为67.0％，与氨曲南联用26例，治疗有效率为69.2％，与多黏菌素B或多黏菌素E联用16例，治疗有效率为68.8％，与碳青霉烯类联用12例，治疗有效率为66.7％。

187例患者中，12例（6.4％）发生不良反应，其中11例发生恶心，其中5例伴呕吐、2例伴上腹痛；1例出现低血钾，予对症治疗后好转。无严重不良反应病例。

## 讨论

血液病患者由于原发性免疫缺陷和接受化疗、放疗、造血干细胞移植等治疗措施导致粒缺，是发生CRE感染的高危人群[4]–[5]，CRO感染的发生率和病死率较其他科室患者更高[6]。《血液肿瘤患者碳青霉烯类耐药的肠杆菌科细菌感染的诊治与防控中国专家共识（2020年版）》指出：在我国粒缺伴发热患者中，能够明确感染部位者占50％左右，最常见的感染部位是肺，其后依次为上呼吸道、肛周、血流等[7]。我国粒缺伴发热患者的致病菌以革兰阴性菌为主，包括大肠埃希菌、肺炎克雷伯菌、铜绿假单胞菌、嗜麦芽窄食单胞菌和鲍曼不动杆菌，病原谱因感染部位和危险因素不同存在差异[8]–[11]。本文纳入病例中检出菌种主要为肺炎克雷伯杆菌（44株，31.9％），其次是铜绿假单胞菌（19株，13.8％），嗜麦芽窄食单胞菌（18株，13.0％），大肠埃希菌（18株，13.0％），阴沟肠杆菌（11株，8.0％）等。CHINET监测网资料显示2014年、2016年、2019年CRE检出率分别为12.5％、22.9％、26.8％，呈上升趋势[11]。本研究病原菌检出138株，CRO 80株（58.0％），CRE 46株（33.3％），病原菌检出率、CRO占比相对较高，与纳入患者多为恶性肿瘤且处于重度粒缺状态相关，部分患者还存在危及生命的血液动力学不稳定。

CAZ-AVI是由头孢菌素类抗菌药物头孢他啶和新型β-内酰胺酶抑制剂阿维巴坦的钠盐组成的固定剂量的复方药物，具有抗多重耐药革兰阴性杆菌产生的多种β-内酰胺酶的活性[13]–[14]。碳青霉烯酶主要包括以下3类：A类丝氨酸β-内酰胺酶、D类丝氨酸β-内酰胺酶和B类金属β-内酰胺酶[15]。文献报道碳青霉烯类耐药大肠埃希菌、碳青霉烯类耐药阴沟肠杆菌以产NDM型金属酶为主（74.8％、52.5％）[16]–[17]，产金属酶菌株可以水解新一代酶抑制剂复合物CAZ-AVI[18]，故CAZ-AVI对产金属-β-内酰胺酶的菌株无活性[19]。本研究检出碳青霉烯类耐药大肠埃希菌病例CAZ-AVI治疗效果亦差，5例检出碳青霉烯类耐药大肠埃希菌的患者，3例死亡；2例加做CAZ-AVI药敏试验患者均提示原发耐药。Falcone等[20]报道产生金属-β-内酰胺酶的肠杆菌引起的血流感染应用CAZ-AVI联合氨曲南治疗后，30 d死亡率（19.2％）明显低于联合其他药物组（44.0％）。国内报道体外实验氨曲南联合CAZ-AVI对产金属酶菌株体外有协同抗菌作用的可能性较高[21]；临床发现CAZ-AVI对于产KPC酶的CRE菌株具有较强的抗菌效果，对产NDM金属酶的CRE菌株无抑制作用，氨曲南联合CAZ-AVI对于产KPC酶和NDM金属酶的CRE菌株均具有良好的抗菌作用[22]。

临床上能更早、更及时的经验性应用高效且不良反应小的抗菌药物抗感染治疗，是减少病死率的关键措施。非CRO组药敏试验提示病原菌对碳青霉烯类及其他抗菌素敏感，但纳入病例在实际应用中多数无效，换用CAZ-AVI治疗后，43例中32例有效，有效率为74.4％，治疗效果良好。经验治疗组均在碳青霉烯类抗菌素或替加环素抗感染效果不佳的时应用CAZ-AVI，有效率为75.4％。文献报道，对于产KPC酶的CRE感染患者，足剂量的CAZ-AVI单药应用临床有效率优于其他抗菌药物的联合治疗方案[23]；荟萃分析显示单药与联用患者微生物清除率和病死率差异均无统计学意义[24]，本研究联合用药患者常为重症感染，亦可能合并病毒、真菌感染的患者，部分患者已存在危及生命的血流动力学异常，合并多脏器功能异常等，多数治疗短期死亡，因此本研究单药和联合用药可能不具有可比性。CAZ-AVI Ⅲ期临床试验证实最常见的不良事件为真菌感染、恶心呕吐、腹痛腹泻、肝损、血小板增多或减少、溶血性贫血等[25]，纳入病例出现相关不良反应发生率6.4％，均症状轻微，患者耐受性及安全性良好。

本研究显示，在CRO患者中，CAZ-AVI的临床有效率较好，不良反应较少且程度轻微，在非CRO及经验性治疗组中，临床有效率更高。血液病患者粒缺合并感染时急需安全有效的抗菌药物治疗，而细菌培养耗时且阳性率有限，临床应用碳青霉烯类抗菌药物及替加环素抗感染效果不佳时，及时有效的治疗至关重要，可经验性应用CAZ-AVI治疗。如病原学证据提示碳青霉烯类耐药大肠埃希菌、碳青霉烯类耐药阴沟肠杆菌感染时，建议联用氨曲南或其他能覆盖产金属酶菌株的抗菌药物协同抗感染。

本研究受回顾性、单中心设计和样本量小的限制，CAZ-AVI在经验性用药的疗效还需与其他抗菌药物进一步对比。
